# Development of postoperative delirium prediction models in patients undergoing cardiovascular surgery using machine learning algorithms

**DOI:** 10.1038/s41598-023-48418-5

**Published:** 2023-11-30

**Authors:** Chie Nagata, Masahiro Hata, Yuki Miyazaki, Hirotada Masuda, Tamiki Wada, Tasuku Kimura, Makoto Fujii, Yasushi Sakurai, Yasuko Matsubara, Kiyoshi Yoshida, Shigeru Miyagawa, Manabu Ikeda, Takayoshi Ueno

**Affiliations:** 1 Division of Health Sciences, Osaka University Graduate School of Medicine, 1-7 Yamadaoka, Suita, Osaka 565-0871 Japan; 2 Department of Psychiatry, Osaka University Graduate School of Medicine, Suita, Osaka Japan; 3 Department of Cardiovascular Surgery, Osaka University Graduate School of Medicine, Suita, Osaka Japan; 4 SANKEN (The Institution of Scientific and Industrial Research), Osaka University, Ibaraki, Osaka Japan

**Keywords:** Medical research, Risk factors

## Abstract

Associations between delirium and postoperative adverse events in cardiovascular surgery have been reported and the preoperative identification of high-risk patients of delirium is needed to implement focused interventions. We aimed to develop and validate machine learning models to predict post-cardiovascular surgery delirium. Patients aged ≥ 40 years who underwent cardiovascular surgery at a single hospital were prospectively enrolled. Preoperative and intraoperative factors were assessed. Each patient was evaluated for postoperative delirium 7 days after surgery. We developed machine learning models using the Bernoulli naive Bayes, Support vector machine, Random forest, Extra-trees, and XGBoost algorithms. Stratified fivefold cross-validation was performed for each developed model. Of the 87 patients, 24 (27.6%) developed postoperative delirium. Age, use of psychotropic drugs, cognitive function (Mini-Cog < 4), index of activities of daily living (Barthel Index < 100), history of stroke or cerebral hemorrhage, and eGFR (estimated glomerular filtration rate) < 60 were selected to develop delirium prediction models. The Extra-trees model had the best area under the receiver operating characteristic curve (0.76 [standard deviation 0.11]; sensitivity: 0.63; specificity: 0.78). XGBoost showed the highest sensitivity (AUROC, 0.75 [0.07]; sensitivity: 0.67; specificity: 0.79). Machine learning algorithms could predict post-cardiovascular delirium using preoperative data.

*Trial registration*: UMIN-CTR (ID; UMIN000049390).

## Introduction

Delirium is a variable acute-onset disturbance of attention and consciousness that has multiple etiologies^1^. Surgery is a common risk factor for delirium, with postoperative delirium occurring in 14–24% of patients following non-cardiac surgery^2,3^. Furthermore, approximately 26–56% of cardiovascular surgery patients who require the use of cardiopulmonary bypass (CPB) develop postoperative delirium^4–7^. Delirium following cardiovascular surgery is independently associated with a decline in cognitive function^8^, activities of daily living (ADL)^6,9,10^ and increased mortality^11^. Thus, postoperative delirium is an important factor that influences patient prognosis.

The American Heart Association issued a statement in 2020 to integrate measures against delirium into Cardiovascular Intensive Care Unit (ICU) care^12^. Recently, the European Society of Anesthesiology (2017)^13^ and POQI-6 (2019)^14^ have introduced guidelines for postoperative prevention and recommended multicomponent non-pharmacological interventions for delirium. The Hospital Elder Life Program (HELP)^15^, the most well-known non-pharmacological multidisciplinary program, reduces delirium by 53%^16^. However, it is difficult to apply these measures to all patients^14^. In a cross-sectional study of acute hospitals implementing HELP, the most common reasons for non-compliance with the original HELP program were lack of adequate staffing and budgetary limitation^17^. As pharmacological therapy, ramelteon and suvorexant also reportedly reduce delirium^18,19^, but premedication to prevent delirium is not recommended for use in all patients^20^. Therefore, early prediction and identification of individuals at high risk for delirium are needed to provide targeted and efficient interventions^14,21^.

Several studies have aimed to predict postoperative delirium. However, the accuracy of prediction models for patients in the ICU is insufficient when applied to those undergoing cardiovascular surgery^22^, and delirium prediction models should be constructed only for a specific group of patients^23^.

Conventional prediction models based on statistical methods for patients undergoing cardiovascular surgery include those by Koster^24^ and Rudolph^4^; both reported an area under the receiver operating characteristic curve (AUROC) of 0.75. Although statistical models such as logistic regression are favorable in terms of model interpretability, machine learning is preferred for prediction models^25^. Clinical prediction models using machine learning algorithms have recently attracted attention. In the area of cardiovascular surgery-associated postoperative delirium, both Mufti^26^ and Xue^27^ showed that the prediction performance of machine learning algorithms was superior to that of conventional statistical models, limited to hyperactive delirium and acute kidney injury-related delirium, respectively. Delirium is classified into three subtypes based on the type of psychomotor activity: hyperactive, hypoactive, and mixed^1^. Hypoactive delirium reportedly accounts for 92% of all cases of delirium in the cardiovascular ICU^28^. Therefore, we must assess and deal with delirium, including the hypoactive subtype which is often overlooked in clinical practice.

We aimed to develop and validate new prediction models for cardiovascular surgery-associated postoperative delirium including hypoactive subtype using machine learning algorithms. Early identification of patients at high risk of delirium is critical to effectively implement prevention strategies, such as multicomponent interventions and prophylactic medications.

## Methods

### Setting and study population

This single-center prospective study included patients aged ≥ 40 years who underwent cardiovascular surgery at Osaka University Hospital between November 2021 and October 2022. The inclusion criteria were patients who underwent coronary artery bypass graft (CABG), valve surgery, ascending aortic replacement (AAR) via a median sternotomy, or minimally invasive cardiac surgery (MICS) using cardiopulmonary bypass (CPB). The exclusion criteria were as follows: (1) patients undergoing transcatheter aortic valve implantation (TAVI) or thoracic endovascular aortic repair (TEVAR); (2) patients managed with deep hypothermic circulatory arrest or selective cerebral perfusion; (3) patients with preoperative delirium; (4) patients diagnosed and treated for dementia preoperatively; (5) patients requiring mechanical ventilation for > 3 days postoperatively, (6) patients with cerebral hemorrhage or stroke within 7 days after surgery; (7) patients requiring reoperation within 7 days after surgery; and 8) patients with suspected alcohol withdrawal delirium. The criteria for alcohol withdrawal delirium were consumption of an average of 60 g or more of alcohol per day immediately prior to admission and the development of delirium within 2 weeks of admission.

### Ethics declarations and consent to participate

This study was approved by the Ethical Review Committee of Osaka University Hospital (No: 21158-3; 2021/09/11) and followed the Declaration of Helsinki. All patients provided written informed consent.

### Variable selection

The electronic medical records of all patients were prospectively reviewed. We collected 38 preoperative factors, including demographics, medical history, laboratory data, and life history, as well as 10 intraoperative factors, including operation time, pump time, and blood fluid balance. The entire list of 48 variables can be found as Supplementary Table S1 and S2 online. A trained nurse visited the patients on any one day from admission to surgery and assessed their function using the Mini-Cog^29^ Japanese version, Geriatric Depression Scale-Short Version-Japanese (GDS-S-J)^30^, and Barthel Index^31^.

### Delirium assessment

All patients were followed for 7 days, with day 0 being the day of surgery. Electronic medical records were reviewed daily. The evaluation of delirium began when the patient was extubated in the ICU. A psychiatrist (MH) or a critical nurse (CN) trained by psychiatrists visited patients after discharge from the ICU and when a change in mental status was suspected, then assess delirium according to the Diagnostic and Statistical Manual of Mental Disorders-5 (DSM-5)^1^. For cases that were difficult to assess, two or more investigators including psychiatrists discussed and came to a final decision. Considering the diurnal variability of delirium, visiting hours were standardized as 3:30 p.m. to 6:30 p.m.

### Data preprocessing and statistical analysis

Some values of the intraoperative propofol dose (25.3%) and HbA1c (1.1%) were missing and thus, these factors were excluded from the analysis. The outliers were used after confirming that they were not erroneously entered. Variables are expressed as median (interquartile range [IQR]) or the number of persons (percentage).

Univariate analysis was used to compare patients who did not develop delirium (non-delirium group) and those who did (delirium group). Student’s t-test was used for continuous variables normally distributed with equal variance, Welch's t-test for variables normally distributed with unequal variance, Mann–Whitney U test for continuous variables not normally distributed, and Fisher's exact test for categorical variables. We used the Shapiro–Wilk test and Bartlett's test to assess data normality and equality of variance, respectively. All were two-tailed tests with a significance level of 5%. Corrections for multiple comparisons were not performed because these analyses were exploratory. R software version 4.0.5 was used for statistical processing.

### Derivation of prediction models

The features used for classification models were selected based on the results of the univariate analysis and the consultations with two skilled cardiovascular surgeons, three psychiatrists, and a nurse. The process of model development is illustrated in Supplementary Fig. S1 online. We compared the performance of classification models using Bernoulli naive Bayes, Support vector machines^32^, Random forest^33^, Extra-trees^34^, and XGBoost^35^. Continuous variables were used as they were. In Bernoulli naive Bayes, continuous variables were binarized to 0 or 1. A binarization threshold is used as a hyperparameter. We searched the hyperparameter spaces of models using a grid search with a fivefold stratified cross-validation. Our dataset is imbalanced because it contains a large proportion of the non-delirium class and a small proportion of the delirium class. Therefore, we used class weights to help the model learn from the imbalanced data. We evaluated the classification performance of these models using a fivefold stratified cross-validation. Seven evaluation metrics were utilized to compare the classification performance of each model: the balanced accuracy, AUROC, area under the precision-recall curve (AUPRC)^36^, sensitivity, specificity, positive predictive value, and F-value. In addition, a predictive model based on the conventional Logistic Regression (LR) method was also built for comparison with the machine learning models. The LR model was validated using the stratified hold-out method. Our experiments were conducted on Python version 3.8.3.

## Result

### Patients and occurrence of delirium

**Figure 1:**
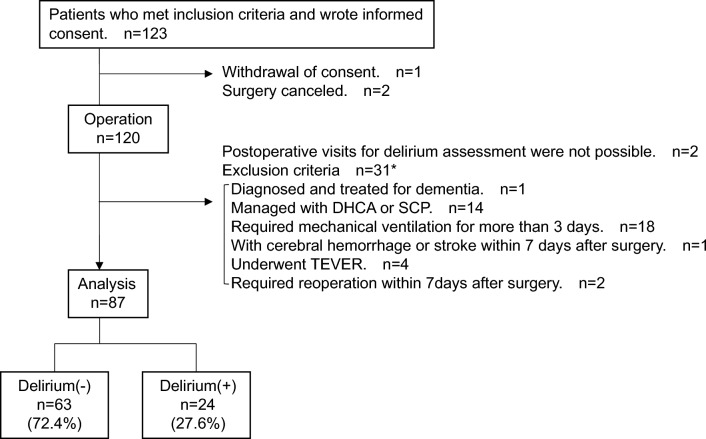
Flow diagram of the study population. DHCA, Deep hypothermic circulatory arrest; SCP, Selective cerebral perfusion; TEVAR, thoracic endovascular aortic repair. *As some patients met more than one exclusion criterion, the total for each criterion does not add up to 31.

Of the 123 patients who met the inclusion criteria and consented to participate in the study, 87 (median age 71 [IQR; 61.5, 75.0] years; 53 [60.9%] males) were included in the analysis. Twenty-four patients (27.6%) developed delirium within seven days after surgery (Fig. 1). Fourteen of the delirious patients assessed delirium during their ICU stay based on electronic medical records, 10 of whom also had delirium symptoms when we visited after discharge from the ICU. No significant difference was found in the length of ICU stay between non-delirium and delirium groups (non-delirium vs. delirium group: median 3 [IQR; 2, 3] days vs. 3 [2, 3], *p* = 0.976). The subtypes included 14 (58.3%) hypoactive, eight (33.3%) mixed, including those who did not present with apparent hypoactivity or hyperactivity, and two (8.3%) hyperactive.

### Risk factors of delirium

The results of the univariate analysis comparing the non-delirium and delirium groups were shown in Supplementary Table S1 and S2 online. Preoperative factors that showed statistically significant differences were age (non-delirium vs. delirium group: median 69 [IQR; 59.5, 74.0] years vs. 74 [69.0, 79.0], *p* = 0.004), use of psychotropic drugs (including sleeping medications) (6 [9.5%] vs. 9 [37.5%], *p* = 0.004), Mini-Cog < 4 (10 [15.9%] vs. 10 [41.7%], p = 0.020), Barthel Index < 100 (3 [4.8%] vs. 5 [20.8%], *p* = 0.034), and CABG (8 [12.7%] vs. 8 [33.3%], *p* = 0.035). No intraoperative factors showed statistically significant differences between the groups.

### Development of prediction models and validation

In the results of the univariate analysis, CABG, which showed *p* < 0.05, was uninterpretable. First, we removed CABG from the set of features for training the models. Second, we selected a history of stroke or cerebral hemorrhage (non-delirium vs. delirium group, 4 [6.3%] vs. 4 [16.7%], *p* = 0.208) and eGFR < 60 (30 [47.6%] vs. 16 [66.7%], *p* = 0.150) as the set of features based on the clinical observation. Consequently, we trained all models using the following features: age, use of psychotropic drugs, Mini-Cog < 4, Barthel Index < 100, history of stroke or cerebral hemorrhage, and eGFR < 60.

**Figure 2:**
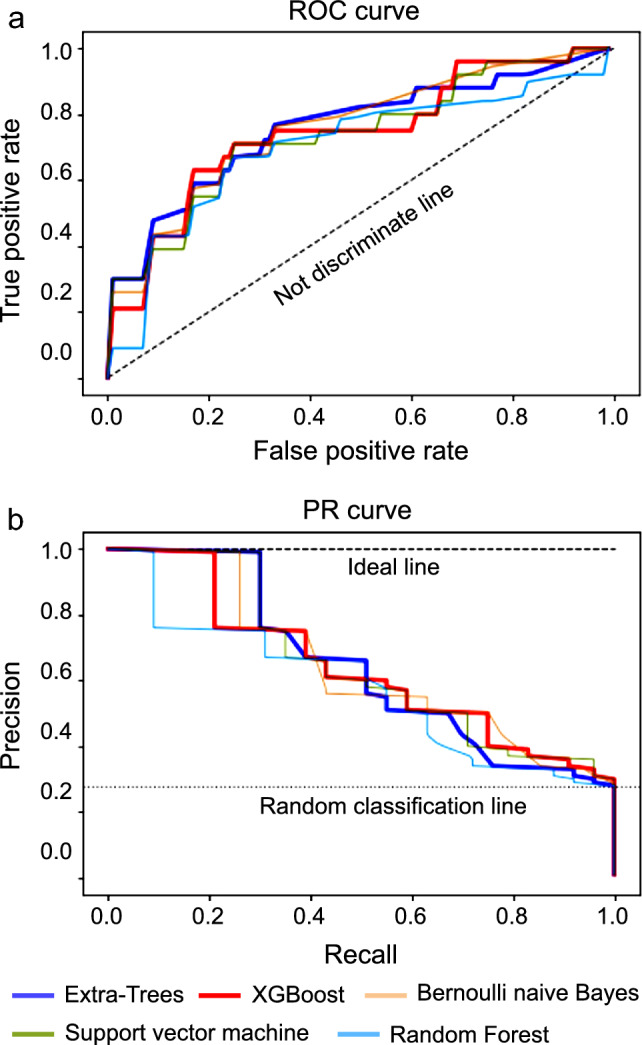
ROC curve and PR curves of the developed models. ROC. curve, Receiver operating characteristic curve; PR curve, Precision-recall curve.

As a result of cross-validation after hyperparameter tuning, the extra-trees model had the best AUROC (0.76 ± 0.11 [standard deviation]) and AUPRC (0.62 ± 0.18), with a sensitivity of 0.63 and specificity of 0.78. XGBoost showed the best sensitivity (AUROC: 0.75 ± 0.07, AUPRC: 0.59 ± 0.17, sensitivity: 0.67, and specificity: 0.79). The conventional LR model showed lower values for the evaluation metrics than other machine learning models. A comparison of the developed models is shown in Table 1 and the ROC and PR curves are shown in Fig. 2.

**Table 1:** Comparison of the performance of the developed models.

Algorithms	AUROC [SD]	AUPRC [SD]	Balanced-accuracy	Sensitivity	Specificity	PPV	F-value
Bernoulli naïve Bayes	0.76 [0.08]	0.59 [0.18]	0.73	0.55	0.94	0.77	0.60
Support vector machine	0.74 [0.08]	0.60 [0.17]	0.74	0.63	0.89	0.68	0.60
Random forest	0.70 [0.15]	0.49 [0.16]	0.68	0.55	0.89	0.56	0.55
Extra-Trees	0.76 [0.11]	0.62 [0.18]	0.70	0.63	0.78	0.52	0.56
XGBoost	0.75 [0.07]	0.59 [0.17]	0.74	0.67	0.79	0.55	0.59
Conventional logistic regression	0.63 [0.15]	0.34 [0.14]	0.68	0.60	0.77	0.50	0.54

AUROC, area under the receiver operating characteristic curve; SD, standard deviation; AUPRC, area under the precision-recall curve; PPV, positive predictive value.

## Discussion

Herein, we developed models to preoperatively predict up to 67% of patients who develop postoperative delirium based on the following six preoperative factors: age, use of psychotropic drugs, Mini-Cog < 4, Barthel Index < 100, history of stroke or cerebral hemorrhage, and eGFR < 60.

Many studies have tried to predict post- cardiovascular surgery delirium based on statistical methods such as logistic regression^4,24,37,38^. However, we applied machine learning to predict postoperative delirium, including the hypoactive subtype. Additionally, we placed importance on the prediction of true-positive patients when developing and validating the models using weighting and PR curves^36^. This resulted in predictive models with similar AUROCs; the sensitivity was more stable than in previous studies. Considering the safety of delirium preventive strategies, such as multicomponent nonpharmacological interventions, a predictive model that emphasizes sensitivity to identify true positives preoperatively may be preferred^39^.

In this study, no intraoperative factors showed statistically significant differences. Hence, we used only preoperative factors to develop the predictive models. Early prediction of postoperative delirium using only preoperative factors enables us to provide efficient interventions for postoperative delirium, such as premedication, preoperative cognitive training, and adequate staffing. For example, the Extra-trees model we built correctly predicted 63% of true positives, while it indicated that 33% of all participants were not targets of predictive strategies preoperatively. Postoperative delirium is influenced by surgical and anesthesia-related factors, such as the use of anticholinergic drugs including opioids^40^ and CPB, which is associated with inflammatory stimuli-induced nerve injury^41^. However, including intraoperative factors in the model reportedly does not improve the prediction accuracy for postoperative delirium^42^. This implies that only preoperative factors. may predict the onset of postoperative delirium, which would allow for the initiation of preoperative prophylactic interventions.

We selected features based on the results of univariate analysis and clinical experiments. A prospective observational study reported that Mini-Cog alone showed discriminative power with an AUROC of 0.77 (95%CI; 0.61–0.93) in predicting postoperative delirium, excluding in cardiovascular surgery^43^. The Mini-Cog is one of the simplest cognitive assessment tools that can be performed in a few minutes^29^. Psychotropic drugs reportedly have side effects on the central nervous system and are risk factors for delirium^44^. Mini-Cog > 4, use of psychotropic drugs, and a history of stroke or cerebral hemorrhage directly reflect the patient's cerebral function. eGFR < 60 is a standard used to evaluate renal dysfunction, which is known to cause brain dysfunction due to overproduction of inflammatory cytokines and hemodynamic changes^45–48^. In older patients, microglial immune responses frequently occur in the central nervous system, and excessive neuroinflammation in response to surgical invasion may lead to postoperative delirium and cognitive dysfunction^49,50^. Some studies cite ADL and instrumental ADL (iADL) impairment as risk factors for delirium^51,52^, and physical function interventions are recommended for preventing delirium^53^. Thus, Barthel Index < 100 was included as a risk factor. Immobility due to impairment of physical function can be related to the decreased cholinergic activity, which is important in the pathophysiology of delirium^54^.

A systematic review in 2021 reported that the most commonly used and best model for predicting delirium in adult inpatients was Random forest, followed by Support vector machine^55^. We used Bernoulli naive Bayes, Support vector machine, Random forest, Extra-trees, and XGBoost and compared the results. Extra-trees and XGBoost, which are superior in terms of AUROC and AUPRC, are ensemble learning models that combine multiple decision trees, similar to that in Random forest. In tree-structured ensemble learning, combining multiple classifiers stabilizes the estimation and suppresses overfitting^56^. Although Random forest did not show particularly good accuracy in this study, such a tree-structured population learning method may be suitable for predicting delirium. The machine learning models we built outperformed conventional LR except for the sensitivity of Random forest and Bernoulli naive Bayes, indicating that machine learning models were better balanced in predicting true positives and true negatives. The advantage of machine learning methods is that they do not have restrictions on their assumption as LR, such as linear distribution of logit transformed values of probability, equivariance of error terms, and the number of predictors^57^. The pathophysiology of delirium is complex and influenced by many factors, machine learning methods, which are highly flexible algorithms, are considered suitable.

Our study has four main limitations. First, some patients with delirium might have been underestimated. Clinical features of delirium are vague, and an estimated 55–70% of hospital delirium cases are missed^58,59^. The incidence of delirium in this study was 27.6%, which is consistent with a previous report indicating the incidence to be 26–56%^4–7,60^ with prospective diagnosis based on DSM-5 criteria. However, delirium occurring in ICU might have been missed because assessments during ICU stays are based primarily on electronic medical records because ICU access was restricted due to the spread of the novel coronavirus infection.

Second, the subjects and sample size were limited by adopting a prospective design at a single center. Although larger sample sizes are generally preferred for building machine learning models, our study included only 87 participants. In addition, our models were validated using stratified 5-fold cross-validation; no external validation was performed. Our results suggest that machine learning may be able to predict post-cardiovascular surgery delirium with greater sensitivity than conventional statistical methods. The developed models could be integrated into a meta-model while expanding the sample size in the future to improve their versatility^23^. In addition, the sample size affects feature selection. Typical feature selection methods in machine learning models include the wrapper and embedding methods that select variables through model training^61^. These methods are used to control model overfitting caused by too many variable dimensions. However, if data used for feature selection is small, there is a risk of causing model overfitting when the method is executed. Thus, unsuitable features for prediction might be selected. Therefore, we applied the statistical feature selection method and discussed it with cardiovascular surgeons, psychiatrists, and a nurse. Machine learning is superior to statistics in terms of discovering data patterns and non-linear relationships^62^, therefore, feature selection methods, such as wrapper and embedding methods, can be applied when we have enough study participants.

Third, the appropriateness of the questionnaire is ambiguous. The mental status of patients aged < 65 years were assessed using the GDS-S-J, which we use in older patients to maintain data consistency. However, the GDS-S-J has been developed specifically for the elderly and thus, its validity in younger adults remains unknown^30^. A questionnaire developed for all age groups should be applied to assess the mental status more accurately.

Finally, we did not include postoperative factors for prediction. Postoperative factors, such as the duration of ICU stay and ventilator support requirement, reportedly affect postoperative delirium^63,64^. However, it is also stated that postoperative factors with unclear temporal relationships to delirium onset should not be used for prediction^23^. If a significant influence of postoperative factors on the development of delirium is suspected in the target population, taking measures such as revision of the exclusion criteria may be required.

## Conclusion

This study’s results suggest that applying machine learning algorithms to predict post-cardiovascular surgery delirium shows superior performance, especially in predicting true-positive patients. The developed models were constructed using only preoperative collectible factors, which enables early identification of patients at high risk of delirium. Clinical applications of such predictive models will contribute to the prevention of development of postoperative delirium in patients undergoing cardiovascular surgery by guiding the implementation of preoperative prevention strategies such as non-pharmacological multifactorial interventions and prophylactic medication that focus on high-risk patients.

### Supplementary Information

Supplementary Information.

## Data Availability

The datasets used and analyzed during the current study are available from the corresponding author upon reasonable request.
