# Author Correction: Development of postoperative delirium prediction models in patients undergoing cardiovascular surgery using machine learning algorithms

**DOI:** 10.1038/s41598-024-51975-y

**Published:** 2024-02-22

**Authors:** Chie Nagata, Masahiro Hata, Yuki Miyazaki, Hirotada Masuda, Tamiki Wada, Tasuku Kimura, Makoto Fujii, Yasushi Sakurai, Yasuko Matsubara, Kiyoshi Yoshida, Shigeru Miyagawa, Manabu Ikeda, Takayoshi Ueno

**Affiliations:** 1 Division of Health Sciences, Osaka University Graduate School of Medicine, 1-7 Yamadaoka, Suita, Osaka 565-0871 Japan; 2 Department of Psychiatry, Osaka University Graduate School of Medicine, Suita, Osaka Japan; 3 Department of Cardiovascular Surgery, Osaka University Graduate School of Medicine, Suita, Osaka Japan; 4 SANKEN (The Institution of Scientific and Industrial Research), Osaka University, Ibaraki, Osaka Japan

Correction to: *Scientific Reports* 10.1038/s41598-023-48418-5, published online 30 November 2023

In the original version of this Article Takayoshi Ueno was incorrectly affiliated with ‘Department of Psychiatry, Osaka University Graduate School of Medicine, Suita, Osaka, Japan’. The correct affiliations are listed below.

Division of Health Sciences, Osaka University Graduate School of Medicine, 1-7 Yamadaoka, Suita, Osaka 565-0871, Japan

Department of Cardiovascular Surgery, Osaka University Graduate School of Medicine, Suita, Osaka, Japan

The original Article has been corrected.

