# Supplementary material for: Development of postoperative delirium prediction models in patients undergoing cardiovascular surgery using machine learning algorithms
Source: Sci Rep. 2023 Nov 30;13:21090. doi: 10.1038/s41598-023-48418-5 (PMC10689441; doi:10.1038/s41598-023-48418-5)
Supplement: Supplementary file 1 — Supplementary Information. [file 41598_2023_48418_MOESM1_ESM.doc]

**Supplementary Information:** Development of postoperative delirium prediction models in patients undergoing cardiovascular surgery using machine learning algorithms by Chie Nagata, Masahiro Hata, Yuki Miyazaki, Hirotada Masuda, Tamiki Wada, Tasuku Kimura, Makoto Fujii, Yasushi Sakurai, Yasuko Matsubara, Kiyoshi Yoshida1, Shigeru Miyagawa, Manabu Ikeda, Takayoshi Ueno

**Table S1:** Preoperative items and comparison in the non-delirium and delirium groups.

|  | **Overall** | **Delirium(-)** | **Delirium(+)** | **P value** |
| --- | --- | --- | --- | --- |
|  | n = 87 | n = 63（72.4%） | n = 24（27.6%） |
| **Patient's characteristics** |  |  |  |  |
| Age(Years), median[IQR] | 71.0 [61.5, 75.0] | 69.00 [59.5, 74.0] | 74.00 [69.0, 79.0] | 0.004 |
| Men, n(%) | 53 (60.9) | 38 (60.3) | 15 ( 62.5) | 1.000 |
| BMI(%), median[IQR] | 22.1 [19.8, 24.2] | 22.1 [19.9, 24.6] | 22.10 [19.5, 22.9] | 0.437 |
| **Operation type** |  |  |  |  |
| CABG, n(%) | 16 (18.4) | 8 (12.7) | 8 ( 33.3) | 0.035 |
| Valve surgery, n(%) | 78 (89.7) | 56 (88.9) | 22 ( 91.7) | 1.000 |
| AAR, n(%) | 6 ( 6.9) | 5 ( 7.9) | 1 ( 4.2) | 1.000 |
| Combined operation of them, n(%) | 28 (32.2) | 17 (27.0) | 11 ( 45.8) | 0.124 |
| MICS, n(%) | 17 (19.5) | 15 (23.8) | 2 ( 8.3) | 0.136 |
| **Assessments** |  |  |  |  |
| Mini-Cog＜4, n(%) | 20 (23.0) | 10 (15.9) | 10 ( 41.7) | 0.020 |
| Barthel Index＜100, n(%) | 8 ( 9.2) | 3 ( 4.8) | 5 ( 20.8) | 0.034 |
| GDS-S-J( score), median[IQR] | 4.0 [2.0, 8.0] | 3.0 [1.5, 7.0] | 5.5 [2.0, 9.0] | 0.123 |
| GDS-S-J≧6, n(%) | 32 (36.8) | 20 (31.7) | 12 ( 50.0) | 0.139 |
| GDS-S-J≧11, n(%) | 2 ( 2.3) | 0 ( 0.0) | 2 ( 8.3) | 0.074 |
| **Medications** |  |  |  |  |
| Number of oral medications brought, median[IQR] | 5.0 [3.0, 8.0] | 5.0 [3.0, 8.0] | 5.5 [2.8, 8.0] | 0.627 |
| Psychotropics, n(%) | 15 (17.2) | 6 ( 9.5) | 9 ( 37.5) | 0.004 |
| Beta-blockers, n(%) | 37 (42.5) | 30 (47.6) | 7 ( 29.2) | 0.149 |
| Statins, n(%) | 23 (26.4) | 18 (28.6) | 5 ( 20.8) | 0.590 |
| Prednisolone, n(%) | 7 ( 8.0) | 6 ( 9.5) | 1 ( 4.2) | 0.668 |
| Furosemide, n(%) | 19 (21.8) | 15 (23.8) | 4 ( 16.7) | 0.571 |
| Nifedipine, n(%) | 4 ( 4.6) | 4 ( 6.3) | 0 ( 0.0) | 0.572 |
| **Medical history** |  |  |  |  |
| Stroke or cerebral  hemorrhage, n(%) | 8 ( 9.2) | 4 ( 6.3) | 4 ( 16.7) | 0.208 |
| Other head diseases, n(%) | 6 ( 6.9) | 5 ( 7.9) | 1 ( 4.2) | 1.0 |
|  | **Overall** | **Delirium(-)** | **Delirium(+)** | **P value** |
|  | n = 87 | n = 63（72.4%） | n = 24（27.6%） |
| Hypertension, n(%) | 35 (40.2) | 24 (38.1) | 11 ( 45.8) | 0.626 |
| Diabetes, n(%) | 20 (23.0) | 13 (20.6) | 7 ( 29.2) | 0.406 |
| PAD, n(%) | 4 ( 4.6) | 1 ( 1.6) | 3 ( 12.5) | 0.062 |
| **Life history** |  |  |  |  |
| Living alone, n(%) | 21 (24.1) | 13 (20.6) | 8 ( 33.3) | 0.265 |
| Never smoker*, n(%) | 53 (60.9) | 36 (57.1) | 17 ( 70.8) | 0.327 |
| Constipation*, n(%) | 7 ( 8.0) | 4 ( 6.3) | 3 ( 12.5) | 0.389 |
| Visual impairment*, n(%) | 60 (69.0) | 46 (73.0) | 14 ( 58.3) | 0.204 |
| Hearing impairment*, n(%) | 18 (20.7) | 10 (15.9) | 8 ( 33.3) | 0.084 |
| Sleep disorder*, n(%) | 22 (25.3) | 15 (23.8) | 7 ( 29.2) | 0.594 |
| **Echo and PWV** |  |  |  |  |
| Carotid artery stenosis, n(%) | 18 (20.7) | 10 (15.9) | 8 ( 33.3) | 0.084 |
| Arteriosclerosis, n(%) | 29 (33.3) | 20 (31.7) | 9 ( 37.5) | 0.620 |
| **Laboratory data** |  |  |  |  |
| eGFR <60mL/min/1.73m2, n(%) | 46 (52.9) | 30 (47.6) | 16 ( 66.7) | 0.150 |
| CRP>1.0mg/dL, n(%) | 21 (24.1) | 14 (22.2) | 7 ( 29.2) | 0.578 |
| Albumin(g/dL), median[IQR] | 4.10 [3.80, 4.40] | 4.20 [3.90, 4.40] | 4.00 [3.50, 4.30] | 0.052 |
| (/nL), median[IQR] | 5.79 [4.79, 6.79] | 6.14 [4.86, 7.19] | 5.25 [4.67, 6.24] | 0.065 |
| Creatinine(mg/dL), median[IQR] | 0.91 [0.74, 1.12] | 0.91 [0.74, 1.11] | 0.92 [0.76, 1.21] | 0.430 |
| Na(mEq/l), median[IQR] | 140.0 [139.0, 142.0] | 140.0 [139.0, 141.5] | 140.5 [138.8, 142.0] | 0.445 |
| ALT(IU/L), median[IQR] | 18.00 [11.00, 22.50] | 18.00 [11.00, 22.00] | 17.00 [11.50, 23.50] | 0.845 |
| Hemoglobin(g/dL), median[IQR] | 13.20 [11.80, 14.40] | 13.10 [11.90, 14.35] | 13.70 [11.40, 14.70] | 0.989 |
| BUN(mg/dL), median[IQR] | 20.00 [16.00, 24.00] | 20.00 [16.50, 23.50] | 18.00 [16.00, 27.50] | 0.992 |
| BNP(pg/ml), median[IQR] | 92.90 [50.05, 299.40] | 91.60 [46.85, 254.65] | 164.95 [54.10, 361.78] | 0.231 |

Note; IQR, interquartile range; BMI, body mass index; CABG, coronary artery bypass grafting; AAR, ascending aortic replacement; MICS, minimally invasive cardiac surgery; GDS-S-J, Geriatric Depression Scale-Short Version; PAD, peripheral arterial disease; PWV, pulse wave velocity; eGFR, estimated glomerular filtration rate ; CRP, C-reactive protein; WBC, white blood cell; ALT, alanine transaminase; BUN, blood urea nitrogen; BNP, brain natriuretic peptide.

p-value: Calculated using Student’s t-test, Welch's t-test, Mann-Whitney U test, or Fisher's exact test.

Mini-Cog: Simple screening tool for dementia.

Barthel Index: An assessment tool for activities of daily living.

Other cerebral diseases: cerebral aneurysm, brain tumor, epidural or subdural hematoma, subarachnoid bleeding, meningitis

* Based on patient self-reports.

**S2:** Intraoperative items and comparison in the non-delirium and delirium groups.

|  | **Overall** | **Delirium(-)** | **Delirium(+)** | **P value** |
| --- | --- | --- | --- | --- |
|  | n = 87 | n = 63（72.4%） | n = 24（27.6%） |
| **Time** |  |  |  |  |
| Operation time(minutes),median[IQR] | 269.0 [219.5, 322.5] | 266.0 [215.5, 322.0] | 270.5 [227.3, 322.0] | 0.890 |
| Cramp time(minutes), median[IQR] | 90.0 [68.0, 114.0] | 89.0 [69.0, 114.0] | 94.5 [65.3, 121.8] | 0.794 |
| Pump time(minutes),median[IQR] | 138.0 [116.0, 185.5] | 139.0 [117.0, 181.0] | 130.0 [115.8, 187.8] | 0.958 |
| **Bleeding and fluid balance** |  |  |  |  |
| Bleeding(ml), median[IQR] | 725.0 [387.5, 1240.0] | 770.0 [471.5, 1240.0] | 615.0 [310.0, 1000.0] | 0.406 |
| Transfusion(ml), median[IQR] | 1240.0 [720.0, 1785.0] | 1210.0 [705.0, 1740.0] | 1380.0 [1000.0, 1930.0] | 0.246 |
| Total balance(ml), median[IQR] | 2087.0 [1312.0, 3026.5] | 2096.0 [1383.5, 3065.0] | 1896.0 [1273.5, 2832.5] | 0.494 |
| **Anesthesia related** |  |  |  |  |
| Fentanyl dose(mg), median[IQR] | 700.0 [600.0, 800.0] | 700.0 [600.0, 800.0] | 700.0 [600.0, 800.0] | 0.537 |
| Remifentanil dose(mg), median[IQR] | 3.6 [2.6, 4.6] | 3.7 [2.5, 4.5] | 3.4 [3.0, 4.9] | 0.743 |
| Sevoflurane use, n(%) | 21 (24.1) | 14 (22.2) | 7 ( 29.2) | 0.578 |
| BIS,median[IQR] | 42.6 [39.2, 46.2] | 43.0 [39.9, 46.9] | 41.6 [38.0, 45.1] | 0.067 |

Note: IQR, interquartile range; BIS, bispectral index

p-value: Calculated using Student’s t-test, Welch's t-test, Mann-Whitney U test, or Fisher's exact test.

**Figure S1**: The process of model development
